# Oral and Intragastric: New Routes of Infection by *Leishmania braziliensis* and *Leishmania infantum*?

**DOI:** 10.3390/pathogens11060688

**Published:** 2022-06-16

**Authors:** Mayra M. Reimann, Eduardo Caio Torres-Santos, Celeste S. F. de Souza, Valter V. Andrade-Neto, Ana Maria Jansen, Reginaldo P. Brazil, André Luiz R. Roque

**Affiliations:** 1 Laboratório de Biologia de Tripanosomatídeos, Instituto Oswaldo Cruz, Fundação Oswaldo Cruz, Rio de Janeiro 21040-360, Brazil; mayrareimann.bio@gmail.com (M.M.R.); jansen@ioc.fiocruz.br (A.M.J.); 2 Laboratório de Bioquímica de Tripanossomatídeos, Instituto Oswaldo Cruz, Fundação Oswaldo Cruz, Rio de Janeiro 21040-360, Brazil; ects@ioc.fiocruz.br (E.C.T.-S.); valter@ioc.fiocruz.br (V.V.A.-N.); 3 Laboratório de Imunomodulação e Protozoologia, Instituto Oswaldo Cruz, Fundação Oswaldo Cruz, Rio de Janeiro 21040-360, Brazil; celcroix@gmail.com; 4 Laboratório de Doenças Parasitárias, Instituto Oswaldo Cruz, Fundação Oswaldo Cruz, Rio de Janeiro 21040-360, Brazil; brazil.reginaldo@gmail.com

**Keywords:** experimental infection, oral transmission, intragastric transmission, hamsters, *Leishmania braziliensis*, *Leishmania infantum*

## Abstract

Although *Leishmania* transmission in nature is associated with the bite of an infected sandfly vector, other possible transmission routes are speculated to occur, such as the oral route. We evaluated the possibility of infection by this route in golden hamsters (*Mesocricetus auratus*) using *Leishmania braziliensis* (Lb) and *Leishmania infantum* (Li). Hamsters were exposed to experimental oral or intragastrical infection with axenic promastigotes, besides oral ingestion of a suspension of cultivated macrophages infected with amastigotes, lesion-fed *Lutzomyia longipalpis,* skin lesion or infective spleen fragment. The parasite’s isolation, besides a positive PCR and IFAT, confirmed the intragastric infection by promastigote parasites. The oral ingestion of macrophages infected with *L. braziliensis* amastigotes was also infective. These results confirmed that *Leishmania* parasites could infect mammals by the intragastric route through the ingestion of promastigote forms (what can happen after a sandfly ingestion) and by the oral ingestion of infected macrophages (what can happen in nature in a predator–prey interaction). The better understanding of these alternative routes is essential to understand their transmission dynamics in nature. As far as we know, this is the first time that oral and intragastric *Leishmania* transmission has been experimentally demonstrated, constituting new infection routes, at least for *L. infantum* and *L. braziliensis*.

## 1. Introduction

Leishmaniosis are widespread infectious diseases with important clinical and epidemiological diversity. The control of this disease is still a worldwide challenge, especially in endemic areas where precarious conditions and social inequality are the main factors that increase the risk of infection [1,2]. *Leishmania* parasites comprise more than 20 species transmitted among hematophagous sandfly vectors and mammalian hosts [3,4,5]. The only proven biological vectors are insects from the order Diptera: Psychodidae, subfamily Phlebotominae, especially *Lutzomyia longipalpis* for *L. infantum*; *Nyssomyia intermedia*, *Ny. neivai*, *Ny. whitmani*, *Migonemyia migonei*, *Psychodopygus wellcomei*, *Psy. complexus* for *L. braziliensis*; *Bichromomyia flaviscutellata*, *Pintomyia nuneztovari* for *L. amazonensis*; and *Bichromomyia* spp., *Nyssomyia* spp., *Lutzomyia* spp., *Psychodopygus* spp., and *Trichophoromyia* spp. for other species of *Leishmania* responsible for tegumentary leishmaniasis in the Americas.

Knowledge about *Leishmania* sp. parasites has advanced in recent decades, but there are still unknown aspects about their transmission mechanisms. In most areas, the low rate of infection in sandflies contrasts with the higher infection rates among mammals, with transmission successfully occurring over time [6,7,8]. Apart from vectorial transmission, the occurrence of alternative infection routes has been recently suggested, especially in areas where cases of leishmaniosis have been reported without the confirmed presence of proven vectors [9,10,11,12] or even the description of any Phlebotominae species [13]. Alternative infection routes already proven, all concerning humans, include (i) blood transfusion [14]; (ii) transplacental/congenital routes [15,16,17]; (iii) needles shared between drug users [18]; and (iv) organ transplantation [19,20]. Sexual transmission was also suggested in dogs, mainly because lesions were found in the reproductive tract of males, and parasites were observed in semen [21,22]. Even transmission by engorged fleas or ticks fed with infected blood and accidentally ingested by dogs or other mammals was already suggested [23,24].

Several parasites from the Trypanosomatidae family can be orally transmitted. The oral route for *Trypanosoma cruzi*, for example, is probably the most important in the wild through the predation of infected mammals or vectors [25,26,27]. *T. dionisii* and *T. c. marinkellei* are bat trypanosomes that are also orally transmitted through the bat predation of *Cavernicola* sp. bugs [28,29,30]. *T. lewisi*, an emerging human trypanosomiasis [31,32], is also successfully transmitted through the accidental ingestion of infected fleas [33,34,35]. Other trypanosomatids are transmitted orally by coprophagy, predation, or even cannibalism [36,37]. Currently, the genus *Leishmania* is divided into four subgenera: *Leishmania*, *Viannia*, *Sauroleishmania*, and *Munidia* [38]. The *Sauroleishmania* subgenus harbors 21 exclusive reptile species, whose most likely transmission route is the oral route. Thus, the *Leishmania*, *Munidia* and *Viannia* subgenera are some of the few Trypanosomatid taxonomic units in which the oral transmission was not yet demonstrated [39,40]. It is worth mentioning that infections in dogs were already reported in nonendemic regions, where no vectors were found, without the parental connection between animals, mating history, or carrying out transfusions and transplants or travel reports to endemic areas. The oral route, through contact with oral mucosa and infected blood during fights or the ingestion of infected ticks, was suggested (but not proved) as a source of infection in these cases [8,23,41,42,43].

This study aimed to evaluate the viability of the oral transmission of *L. braziliensis* and *L. infantum* in the golden hamster (*Mesocricetus auratus*), recognized as the most susceptible experimental model [44,45], in a controlled environment. We hypothesize that *L. (Viannia)* and *L. (Leishmania)* promastigote and amastigote forms can infect when ingested by a susceptible mammal host.

## 2. Results

Promastigote forms of Leishmania parasites, at least the most widespread species in Brazil, *L. braziliensis* and *L. infantum*, can infect mammalian hosts when intragastrically inoculated. The infection was confirmed by parasite isolation and positive PCR in skin, spleen, and liver samples, besides positive IFAT. Macrophages infected with *L. braziliensis* amastigotes also infect hamsters when orally ingested, as demonstrated by both positive PCR and IFAT (Table 1).

### 2.1. Leishmania braziliensis

The animals intradermically inoculated with *L. braziliensis* in the left front paw (control group) showed the characteristic lesions of cutaneous leishmaniosis, such as nodular lesions of the skin that increased until become ulcerated in the center. This happened approximately 60 days after the experimental infection. The euthanasia was performed 64 days post infection—dpi.

**Figure 1:**
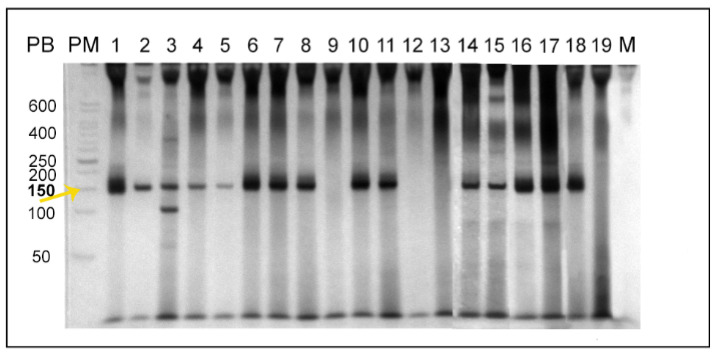
Polyacrylamide gel electrophoresis of the multiplex PCR products aiming to detect *Leishmania* sp. DNA. (PM) 50 bp ladder; (1–8) represent positive samples from the 2 animals in group Lb1: 1 and 6 sites of inoculation, 2 and 5 abdominal skin, 3 and 8 liver, 4 and 7 spleen; (9–13) represent samples from group Lb2, being animal 1, the hamster in which *L. braziliensis* were reisolated: 9 abdominal skin, 10 spleen, 11 liver of animal 1; 12 abdominal skin, 13 spleen of animal 2; (14–17) represent positive samples from the 2 animals in group Li2: 14 and 16 spleen, 15 and 17 liver; (18) Positive control; (19) Negative control; (M) Mix PCR. The arrow and the positive sign indicate the expected size of base pairs (between 120–145 bp).

Parasites were isolated from the inoculation site, spleen, and liver from two infected hamsters (Lb1), and the isolated parasites were amplified and cryopreserved after two in vitro passages. Amastigotes were observed in the lesion, spleen, and liver imprints. Although amastigotes were not observed in the histology, a large amount of inflammatory infiltrate was observed in the liver of both animals. Both animals were positive in serology, showing a titration of 1:320 in the IFAT. *L. braziliensis* DNA was also detected through multiplex PCR in both animals (Figure 1).

**Figure 2:**
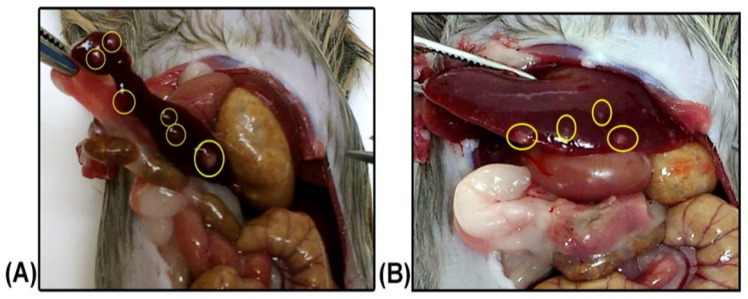
Nodular lesions suggestive of *Leishmania* sp. infection. Spleen (**A**) and liver (**B**) from one of the animals of group Lb2 intragastrically inoculated with *L. braziliensis*.

The animals intragastrically infected with *L. braziliensis* were followed up weekly for 6 months, and no apparent clinical changes were observed. However, at necropsy, in one of them, it was possible to observe nodular, pale, crusted lesions suggestive of *Leishmania* sp. infection in the liver and spleen (Figure 2).

**Figure 3:**
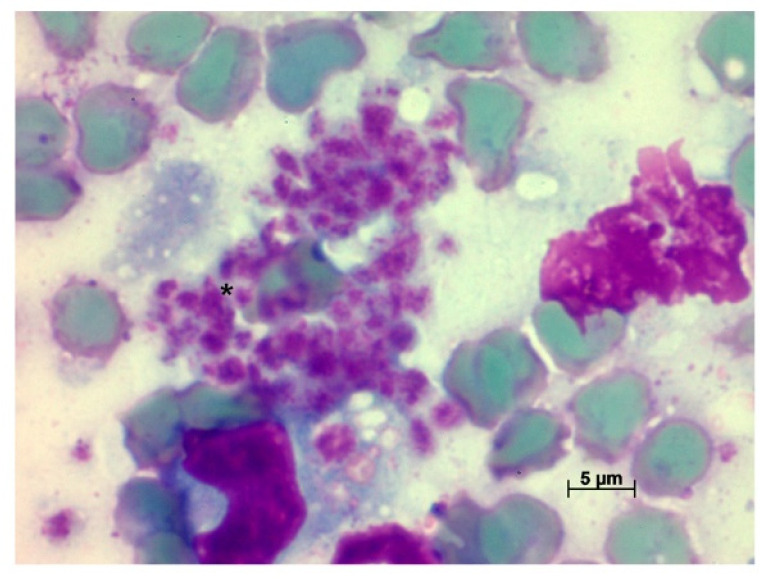
Amastigotes (*) present in spleen imprint. One of the animals in group Lb2 was inoculated intragastrically with 10^6^ promastigote forms of *L. braziliensis*.

It was possible to isolate the parasites in the spleen and liver from the same hamster in which nodular lesions were observed. The infection of this animal was also confirmed by positive PCR in spleen and liver samples (Figure 1) and amastigotes were observed in the spleen imprint slides (Figure 3). Although positive parasitological results were observed only in one of the hamsters, both became infected after the intragastric inoculation of *L. braziliensis*, as demonstrated by the positive serology, presenting a serological titre of 1:160 (Table 1). The histological examination of the spleen and liver of both animals in this group did not result in amastigote detection; however, it was possible to observe the presence of inflammatory infiltrates in the liver of both animals.

Animals orally infected with cultures of macrophages infected with *L. braziliensis* in the Lb4 group were followed up weekly for 6 months, with no clinical changes. In these animals, positive results in multiplex PCR of the spleen, liver, and stomach, in addition to a titre of 1/320 in the IFAT (Table 1). All other hamsters that comprised the experimental groups Lb3 and Lb5–Lb7 were followed up for six months and no positive results were observed.

### 2.2. Leishmania infantum

The control group (Li1) was intraperitoneally inoculated with *L. infantum* and followed up for six months. At 180 dpi, one of the animals presented a visible increase in the abdomen and difficulty feeding. In the necropsy, visible splenomegaly was confirmed. The infection was confirmed in both animals through *L. infantum* isolation in skin, spleen, and liver fragments. These cultures were amplified and cryopreserved after 2–3 in vitro passages. Both animals were also positive by PCR of the abdominal skin, liver, and spleen, besides IFAT (titre of 1/160). No positive results were observed in imprinting, and histology was not performed (Table 1).

Two hamsters intragastrically inoculated with *L. infantum* (Li2) became infected and parasites were isolated from skin, spleen, and liver fragments. Positive PCRs were obtained from the same tissues and a positive IFAT titre of 1/40 was demonstrated (Table 1, Figure 1). No positive results were observed in hamsters infected with *L. infantum* in the other experimental groups.

## 3. Discussion

Our results corroborate the hypothesis of the existence of alternative routes for *L. infantum* and *L. braziliensis* infection in hamsters, which are the most widespread *Leishmania* species in Brazil. These infections were confirmed by the parasites’ isolation in axenic cultures, in addition to positive results in the Multiplex PCR and serology. This is the first time that the intragastric and oral transmission in *Leishmania* has been demonstrated. Before that, some studies had already suggested the possible occurrence of these routes, mainly in studies involving dogs. In these works, the authors hypothesized that the transmission occurred due to fights, given the presence of skin lesions and contact with the oral mucosa of another dog, or by the ingestion of ticks that had just been fed with blood contaminated with the parasite. In all these cases, canine infection occurred without the presence of proven vectors in the area and after all other recognized transmission routes (transplacental, transfusion and sexual) were ruled out [23,41,42,43].

Although someone can initially consider that the intragastric infection is an artificial transmission route, it can also be hypothesized that this route may occur in nature through the ingestion of infected vectors, considering the cases in which the digestion of that insect starts only in the stomach of the host. This ingestion can occur accidentally when a mammal scratches itself with its mouth and may swallow an infected sandfly or even ingest other hematophagous arthropods that have previously ingested infected blood from another mammal. Accidental ingestion may also occur in cases where a mammal realizes that a phlebotomine insect is flying nearby and decides to capture it. This ingestion can also occur in mammals that include insects in their diet [46,47]. Mostly, due to their tiny size, an infected insect will hardly be chewed, ending up being swallowed whole. Considering the Carnivora, which include some of the most reported *Leishmania* host species, most of them swallow their prey in large pieces under an incomplete chewing process [48]. This eating habit facilitates the ingestion of fragments of infected tissues, which are directed to the stomach, where most of the digestion begins, reproducing the intragastric route described herein for infected macrophages. It is recognized that naturally infected small mammals usually present *Leishmania* parasites in spleen and liver fragments, including parasites from both the *Leishmania* and *Viannia* subgenus [5]. In this sense, the intragastric, and not the oral, route seems to be better reproduced in nature when a carnivore species prey on an infected mammal.

The golden hamster (*M. auratus*) is considered the best and most susceptible model to a variety of intracellular parasites, including *Leishmania* sp. [49]. Moreover, male hamsters were demonstrated to present higher susceptibility to infection by *L. (V.) panamensis*, with more extensive and more severe lesions and a greater parasitic burden on the lymph nodes than females [50]. Since then, most studies, including this, have used male golden hamsters as experimental models. In cases of the traditional route of infection, the clinical symptoms of cutaneous leishmaniosis usually appear between 30 days and 4 months (intradermal), and the clinical signs of visceral leishmaniosis typically manifest later, within 3 to 6 months (intraperitoneal) in hamsters [44,51,52]. This is the reason for the different periods of follow up adopted for hamsters infected by *L. braziliensis* (4 months) or *L. infantum* (6 months).

The viability and infectivity of the THOR strain (*L. braziliensis*) were attested by the intradermic inoculation of parasites, which resulted in the expected clinical symptoms, such as skin lesions at the inoculum site followed by positivity in diagnostic tests: tissue cultures, imprints, PCR, and serology. For hamsters intraperitoneally infected with *L. infantum* (the control group), we defined a maximum of 6 months for follow-up. It is known that among the main clinical symptoms of VL are splenomegaly, anemia, weight loss, alopecia, keratoconjunctivitis, and paresis of the posterior limbs [51]. After six months of infection, it was possible to observe an increase in the abdomen of one of the hamsters, which was further characterized as splenomegaly in the necropsy, in addition to a loss of appetite. The infection was further confirmed by parasite isolation, besides positive PCR and serology.

Surprisingly, the success of infection when parasites were orally administered was not the same when intragastrically inoculated. One factor that may have influenced this difference is the host’s saliva, which can play a key role in combating parasites [53,54]. It is possible that the defense mechanisms present in hamster’s saliva have managed to eliminate the infection. Despite the slight contact between saliva and parasite culture in the animals’ mouths, since it quickly swallowed the inoculum, the protective action of hamsters’ saliva has already been demonstrated in *Leptospira* sp. after experimental infection carried out in a similar manner as performed here. In addition, the presence and action of saliva is not restricted to the oral cavity and is also present in other parts of the gastric tract up to the stomach. Even if the hamster’s saliva may have had a protective action against the establishment of the oral infection, we cannot exclude the possibility to occur in other mammals. The saliva’s chemical composition varies according to the mammal species involved, as already observed when Asoh and coauthors compared the properties of the saliva of hamsters and humans [54].

Animals from the Lb5 group ingested sandflies that had fed directly in the skin lesion from hamsters in which we isolated *L. braziliensis*, but these hamsters did not become infected. It is worth mentioning that, despite not being associated with *L. braziliensis* transmission in the wild, the permissibility of *L. longipalpis* to different *Leishmania* species, including *L. braziliensis*, was already demonstrated [55,56]. The total number of sandflies used in this work (*n* = 20), apparently low in comparison with other studies of infection in sandflies [57,58], was not so small considering that only the injured paw was exposed to the vectors. Nevertheless, only six females fed and survived the incubation period. Given this low number, we chose to offer all of them to feed the hamsters (three insects for each), making it impossible to carry out a direct examination or PCR to confirm the establishment of the infection. Thus, we should consider the possibility that these phlebotomies were not infected, or at least not with enough parasites to infect the hamsters, and this infection route cannot yet be discarded to occur in nature.

Other experimental groups of *L. braziliensis* were inoculated with a fragment of the dermal lesion (Lb6) and spleen fragment (Lb7) containing macroscopic lesions of infected hamsters. These infection pathways were conceived to reproduce what can happen in nature in a predation event. *Leishmania* amastigotes are present in nests inside host tissues and are not homogeneously distributed among tissues or even within the same tissue. Thus, it is expected that within the same tissue, there are fragments that contain many parasites and others with little or no amastigote nest [59]. It was already observed that 30% of the samples evaluated during the experimental infection of the wild *Thrichomys* sp. rodent species displayed different results for the same tissues [60]. Alternatively, it is possible that, although positive, the number of amastigotes present in these fragments was not enough for the infection establishment in the hamsters [61]. Moreover, in nature, a predator certainly ingests a large amount of tissue during its feeding, not just small fragments.

Hamsters from the Lb4 group became infected after ingesting a suspension of in vitro murine macrophages infected with *L. braziliensis*, as observed by the positive PCR and serology with a high titre of 1:1280. These results confirmed the feasibility of infection by the oral route. In nature, this type of infection can occur at a time of predation among carnivorous mammals, such as canids, which do not have the habit of chewing food a lot swallowing large pieces of meat [62,63] that may be infected with *Leishmania* amastigotes. Similar results were not observed in the Li4 group, using macrophages infected with *L. infantum* parasites. We attribute this to some peculiarity of the strain, or even to *Leishmania* species. More tests are needed, improving parasite load, including other *L. infantum* strains, and increasing the number of inoculated animals before ruling out the possibility that this infection route occurs in *L. infantum* transmission cycles.

Aiming to collaborate with the movement to increase intellectual humility in research articles [64], it is important to discuss some potential limitation of the present study, as the number of animals used. It is worth mentioning that we did not aim to compare the efficacy of distinct transmission routes or to propose new experimental approaches in leishmaniosis, but to demonstrate that the oral route is a possible infection pathway to be considered. Moreover, to prove this unprecedented discovery, one single positive individual is enough, especially considering that an outbreed experimental model was employed. We proved this using *L. braziliensis* and *L. infantum* promastigotes from axenic culture, and macrophages infected with *L. braziliensis* amastigotes. The intragastric inoculation was performed by an experienced handler and no injury in the gastric tract was observed. Even with we consider the remote hypothesis that the infection was established because of a tissue injury caused by the gavage needle, the infection of the hamsters that received an oral suspension of infected macrophages demonstrate the success of the oral infection route. On the opposite way of the positive results, the negative results are seriously impacted with the low number of animals per group. Because of that, we did not discard any of this transmission routes, but opted to include them in the manuscript to encourage other authors to repeat these experiments using different animal models, higher number of animals, and distinct parasite strains. We recognize that these negative results may be biased, but the positive ones, which confirmed our hypothesis, are sound.

Knowing all possible ways of transmitting a parasite is of fundamental importance to understand its relationship with the environment, vectors, and hosts and, therefore, propose effective preventive and control strategies. Our results proved that promastigote and amastigote forms of *L. braziliensis* and *L. infantum* can infect *M. auratus* when intragastrically or orally administered. This points to two possible new infection routes in mammalian leishmaniosis and highlights the importance of more in-depth and detailed studies to evaluate the viability of the oral transmission of *Leishmania* species. Furthermore, the intragastric and oral transmission routes described in an unprecedented way in this work should be considered possible alternative forms of *Leishmania* sp. infection and/or transmission.

## 4. Materials and Methods

### 4.1. Animals

Twenty-eight golden hamsters (*Mesocricetus auratus*) derived from the Institute of Science and Technology in Biomodels (ICTB) Oswaldo Cruz Foundation were used. These animals were 3 to 4 weeks old, males, with approximately 30 g. They were maintained under controlled temperature during complete follow-up and provided with appropriate food and filtered water ad libitum.

### 4.2. Parasites and Infection

The *Leishmania* strains used were the *L. braziliensis* (MCAN/BR/98/R619) strain THOR and *L. infantum* (MHOM/MA/67/ITMAP-263). These samples were kept cryopreserved in liquid nitrogen (−196 °C) or in NNN (Nicolle, Novy, McNeal) medium with Schneider liquid overlay at 28 °C. The hamsters were split into 11 experimental groups (Table 2).

Experimental infections were always carried out using 1 × 10^6^ promastigotes in the stationary phase of parasite growth derived from axenic cultures with a maximum of 4 passages.

The hamsters were restrained, and the final culture was inoculated orally with a needleless syringe, administering the culture directly into the animal’s mouth. The intragastric inoculation was performed using a syringe with a gavage needle, which was inserted into the animal’s mouth and through the esophagus until reach the stomach where the culture was released. This procedure was performed by an experienced handler.

After infection, the hamsters were followed up for a maximum of 6 months, when they were euthanized to collect blood and tissue fragments: abdominal skin, spleen, liver, and site of inoculation (this latter, only for Lb1). The follow-up period was shorter, and necropsy was anticipated in the groups where the animals had clinical changes that could represent a risk to the animal’s life.

### 4.3. Obtaining Infected Macrophages

To accomplish macrophage infection, cultures of *Leishmania* spp. were kept at 26 °C in Schneider^®^ culture medium (Sigma-Aldrich Corp., St. Louis, MO, USA) supplemented with 20% foetal bovine serum (SFB).

Murine peritoneal macrophages were collected with RPMI medium (Sigma-Aldrich Corp., St. Louis, MO, USA) supplemented with 10% foetal bovine serum, 1% pyruvate, 1% glutamine, 1% penicillin, and streptomycin (Gibco, Thermo-Fisher Scientific Inc., Waltham, MA, USA). The macrophages were plated in Petri dishes (2.0 × 10^6^ cells/mL) together with a coverslip for later counting of infected cells. Promastigotes (five parasites per macrophage) at the stationary phase were added to macrophage cultures and incubated for 4 h at 37 °C (*L. infantum*) and 34 °C (*L. braziliensis*) in 5% CO_2_ atmosphere. After the incubation time, the cultures were washed to remove the noninternalized parasites. For *L. infantum*, an additional step was performed: RPMI medium was added to the culture with 2% horse serum and incubated overnight with 10% bovine serum.

The cultures were maintained at 37 °C with 5% CO_2_ for 72 h when the coverslip was removed from the plate to quantify amastigotes inside macrophages. Then, the cultures were washed with PBS, and the cells were harvested using a cell scraper. The final culture was inoculated in the hamsters by the oral route (with a needleless syringe, directly into the animal’s mouth) considering 10^6^ parasites in a volume of 0.15–0.3 mL.

### 4.4. Lutzomyia Longipalpis Feed on Infected Hamsters

The infected hamsters were anesthetized, and only the damaged area in the front paws of hamsters intradermically infected with *L. braziliensis* (Lb1) was exposed to *L. longipalpis* sandflies. It was used on 20 females aged approximately 7–10 days for 1 h. After this period, the engorged sandflies (*n* = 9) remained in the BOD (Biochemical Oxygen Demand) greenhouse for four days receiving a sugar solution containing antibiotics. On the fifth day, the six surviving females were immobilized by the cold and put inside hamsters’ mouth (three to each), which actively and rapidly swallowed the insects.

### 4.5. Euthanasia and Sample Collection

The hamsters were anesthetized with ketamine hydrochloride (100 mg/mL) associated with acepromazine (10 mg/mL) at necropsy. Then, blood was collected through a cardiac puncture. After blood collection, the hamsters were euthanized with Tiopental (100 mg/kg) and transferred to a class II biological safety cabinet where the following procedures were performed with the collected fragments of spleen, liver, abdomen skin, and inoculation site (only for Lb1): (i) tissue imprints; (ii) storage in saline with antibiotics (350 IU penicillin and 150 mg/mL streptomycin (Sigma^®^, St. Louis, MO, USA); (iii) storage in 1.5 mL tubes containing ethanol at −20 °C for molecular diagnosis; (iv) storage in PFA (paraformaldehyde) at 4% in PBS 0.01 M, pH 7.45 for histopathological diagnosis (only performed on animals inoculated with *L. braziliensis*). The collected blood was centrifuged, and serum was stored at −20 °C for serology.

### 4.6. Tissue Imprints

Tissue imprints were fixed with methanol and Giemsa stained [65,66]. Imprints were observed using immersion oil in an Olympus Carl Zeiss Axiostar Plus^®^ Light Microscope at 1000× magnification.

### 4.7. Cultures

Tissue fragments collected in saline were stored at 4 °C for 24 h. Then, they were transferred to culture tubes containing NNN and Schneider liquid medium. They were kept in a BOD chamber at 28 °C and monitored twice a week. Negative samples were followed for up to 1 month and then discarded. When positive, cultures were amplified and cryopreserved.

### 4.8. Multiplex PCR for the Detection of Leishmania *sp.* kDNA

DNA extraction of tissues collected in absolute ethanol was performed using the commercial Wizard Genomic DNA Purification Kit (Promega, Madison, WI, USA) following the manufacturer’s instructions after an initial rehydration step [67]. Positive and negative controls were used in each reaction, from DNA extraction to electrophoresis.

Multiplex polymerase chain reaction (PCR) was performed using the commercial PCR pureTaq Beads kit (illustrates PuReTaq Ready To Go PCR Beads^®^, GE Healthcare, Chicago, IL, USA), and primers targeting the conserved region of *Leishmania* sp. kDNA minicircle: 150F (5′-GGG (G/T)AGGGGCGTTCT(C/G)CGAA-3′) and 152R (5′-(C/G)(C/G)(C/G)(A/T)CTAT (A/T)TTACACCAAC CCC-3′); and a conserved region of the mammalian GAPDH gene: 212F (5′-ACC ACAGTCCATGCCATCAC-3′) and 212R (5′-GTCAGGTCCACCACTGACAC-3′) [68]. The reactions were carried out as follows: 95 °C for 1 min, 30 cycles of 95 °C for 30 s, 61 °C for 30 s, and 72 °C for 30 s, followed by a final extension of 72 °C for 1 min. In addition, the PCR products were subjected to electrophoresis in 8% polyacrylamide mini gels revealed by silver nitrate using the PlusOne DNA Silver Staining^®^ kit (GE Healthcare, Chicago, IL, USA). The images of the gels were documented with a GS-800^®^ densitometer (Bio-Rad, Hercules, CA, USA).

### 4.9. Serological Diagnosis

The serological diagnosis was performed by investigating the level of IgG antibodies through the Indirect Immunofluorescence Assay (IFAT) [69] using the commercial hamster’s anti-IgG conjugated to Sigma^®^ fluorescein Isothiocyanate (Sigma Aldrich, St. Louis, MO, USA). Serum was serially diluted from 1:10 to 1:320, and the employed antigen was an equal mixture of whole parasites of strains 566 and 579 of *L. braziliensis* or *L. infantum*, respectively [70]. The reaction was observed under a light microscope with an immunofluorescence system of a 12 V50 W mercury lamp (Osram, Munich, Germany) at 400× magnification. All reactions with positive and negative controls were performed in duplicate, and the adopted cut-off was 1:10 [25].

### 4.10. Histopathological Diagnosis

The fragments were fixed in 4% PFA for at least 72 h and cleaved in an automatic tissue processor (Leica^®^—TP 1020). After standard processes of dehydration and diaphanization in xylol, fragments were embedded in paraffin in an inclusion station (Microm^®^—AP 280). Five micrometer sections were obtained in a rotating microtome (Microm^®^—HM 360) and stained by the hematoxylin-eosin technique. Two slides were assembled per tissue (after discarding the first slides) and entirely observed under microscopy analysis using a Zeiss-Axioplan microscope and photographed with Axioplan 2^®^.

## Figures and Tables

**Table 1:** Results of experimental groups of hamsters inoculated with *Leishmania* spp.

Group	Route of Infection	Culture (Tissue)	Imprint (Tissue)	Serology (Titre)	PCR (Tissue)	Histology
Lb1	Intradermal (Control Group)	Positive (site of inoculation, spleen, and liver)	Positive (site of inoculation, spleen, and liver)	Positive (1/320)	Positive (site of inoculation, intact skin, spleen, and liver)	Negative
Lb2	Intragastric	Positive (spleen and liver)	Positive (spleen)	Positive (1/160)	Positive (spleen and liver)	Negative
Lb3	Oral—Axenic culture	Negative	Negative	Negative	Negative	Negative
Lb4	Oral—Culture of macrophages (*L. braziliensis*)	Negative	Negative	Positive (1/1280)	Positive (stomach, spleen, and liver)	ND *
Lb5	Oral—Sandflies	Negative	Negative	Negative	Negative	Negative
Lb6	Oral—Fragment of dermal lesion	Negative	Negative	Negative	Negative	Negative
Lb7	Oral—Fragment of spleen	Negative	Negative	Negative	Negative	Negative
Li1	Intraperitoneal (Control Group)	Positive (intact skin, spleen and liver)	Negative	Positive (1/160)	Positive (intact skin, spleen and liver)	ND *
Li2	Intragastric	Positive (spleen)	Negative	Positive (1/40)	Positive (intact skin, spleen and liver)	ND *
Li3	Oral—Axenic culture	Negative	Negative	Negative	Negative	ND *
Li4	Oral—Culture of macrophages (*L. infantum*)	Negative	Negative	Negative	Negative	ND *

ND * = not done.

**Table 2:** Groups of hamsters experimentally inoculated with *Leishmania braziliensis* (Lb) or *L. infantum* (Li).

Group	Route of Infection	Infected Hamsters (*n*)
Lb1	Intradermal (Control Group)	2
Lb2	Intragastric—Axenic culture	2
Lb3	Oral—Axenic culture	2
Lb4	Oral—Culture of macrophages infected with *L. braziliensis*	2
Lb5	Oral—*Lutzomyia longipalpis* fed directly on skin lesions from animals of group Lb1	2
Lb6	Oral—Fragment of dermal lesion from animals of group Lb1	2
Lb7	Oral—Fragment of spleen with macroscopic lesions from an animal of group Lb2	2
Li1	Intraperitoneal (Control Group)	2
Li2	Intragastric—Axenic culture	6
Li3	Oral—Axenic culture	6
Li4	Oral—Culture of macrophages infected with *L. infantum*	2

## Data Availability

Not applicable.
